# The Expression Pattern of the Splice Variants of Coxsackievirus and Adenovirus Receptor Impacts CV-B3-Induced Encephalitis and Myocarditis in Neonatal Mice

**DOI:** 10.3390/ijms26157163

**Published:** 2025-07-24

**Authors:** Xinglong Zhang, Xin Zhang, Yifan Zhang, Heng Li, Huiwen Zheng, Jingjing Wang, Yun Liao, Li Yu, Dandan Li, Heng Zhao, Jiali Li, Zihan Zhang, Haijing Shi, Longding Liu

**Affiliations:** 1 Institute of Medical Biology, Chinese Academy of Medicine Sciences, Peking Union Medical College, Kunming 650031, China; zhangxinglong@imbcams.com.cn (X.Z.); kaimenfanggou@126.com (X.Z.); zhangyifan@imbcams.com.cn (Y.Z.); liheng@imbcams.com (H.L.); zhenghuiwen@imbcams.com.cn (H.Z.); wangjingjing@imbcams.com.cn (J.W.); liaoyun@imbcams.com.cn (Y.L.); yuli@imbcams.com.cn (L.Y.); lidandan@imbcams.com.cn (D.L.); zhaoheng@imbcams.com.cn (H.Z.); lijiali@student.pumc.edu.cn (J.L.); zzh@student.pumc.edu.cn (Z.Z.); haijingshi@hotmail.com (H.S.); 2 Key Laboratory of Systemic Innovative Research on Virus Vaccine, Institute of Medical Biology, Chinese Academy of Medical Sciences, Peking Union Medical College, Kunming 650118, China

**Keywords:** CV-B3, CAR, spatial and temporal expression, susceptibility, inflammatory pathology

## Abstract

Coxsackievirus B3 (CV-B3) infection causes inflammatory conditions such as viral myocarditis and meningitis, and incidence rates are rising annually. While children are more likely to be affected by severe manifestations, the molecular basis of this age-dependent susceptibility is poorly understood. In this study, we used young Balb/c mice at three developmental stages (7-, 14-, and 30-day-old mice) to investigate CV-B3 pathogenesis. Our findings revealed that 7-day-old mice exhibited substantial infection susceptibility and pathological severity compared to older mice. Critically, an age-dependent analysis showed a progressive decline in the expression of CV-B3-binding Coxsackievirus and Adenovirus Receptor (CAR) splice variants (CAR1 and CAR2) at both the transcriptional and translational levels as the mice matured from 7 to 30 days. These receptor isoforms demonstrated a direct correlation with viral replication efficiency in younger hosts. Concurrently, aging was associated with a rise in non-binding CAR variants (CAR3 and CAR4). During CV-B3 infection, the abundance of CAR1/CAR2 in young mice facilitated accelerated viral proliferation, coupled with the hyperactivation of the NLRP3 inflammasome and the expansion of IL-17-producing γδT cells (γδT17 cells). This cascade triggered excessive production of proinflammatory cytokines (IL-1β, IL-18, and IL-17), culminating in pronounced inflammatory infiltrates within cardiac and cerebral tissues. These findings establish NLRP3 inflammasome dysregulation as a critical determinant of CV-B3-induced tissue damage and provide novel insights into the heightened susceptibility to CV-B infection during early life and its associated severe disease rates.

## 1. Introduction

Hand, foot, and mouth disease (HFMD) remains a critical public health challenge in China, consistently ranking within the top three Class C infectious diseases in terms of both incidence and mortality [1,2,3]. While the deployment of inactivated EV-71 vaccines has mitigated EV-71-associated HFMD outbreaks [4,5], the prevalence of non-EV-A71 pathogens is increasing year by year [6]. Many enteroviruses can cause HFMD, but, in general, coxsackieviruses are important pathogens. In addition to coxsackievirus group A (CV-A), coxsackievirus B3 (CV-B3) has also emerged as a notable etiological agent of HFMD. CV-B3 infections manifest in various clinical presentations, including blisters and HFMD-like rashes, as well as causing myocarditis and encephalitis in children in particular [7].

The severity of CV-B infections in younger populations is particularly concerning [8,9,10]. Several lines of epidemiological evidence support this age-specific vulnerability affecting young children, indicating an age-related distribution of CV-B infections, and high mortality rates have been observed in newborns and young children infected with CV-B2 (11.1%), CV-B3 (18.8%), and CV-B4 (40%) [11]. Numerous clinical studies have consistently reported that CV-B infections in infants are frequently accompanied by severe clinical symptoms and poor outcomes [6,9,11]. Although CV-B3-related myocarditis and encephalitis have been documented in pediatric populations, the molecular mechanisms underlying the age-specific susceptibility to these severe manifestations remain poorly understood. While this heightened vulnerability was traditionally attributed to immature immune systems in young children, this explanation appears to be oversimplified given the paradoxical observation that the same demographic exhibits a markedly reduced susceptibility to other viral pathogens, such as SARS-CoV-2 [12,13,14], suggesting that age-dependent susceptibility patterns may be governed by pathogen-specific factors rather than generalized immune immaturity. From another viewpoint, the coxsackievirus and adenovirus receptor (CAR) serves as the primary cellular entry point for CV-B viruses, and it has been identified as a critical determinant of human infection susceptibility [1]. The CAR’s pronounced tropism, particularly in cardiac tissues, proves that it is essential not only for the direct infection of target organs but also for viral dissemination from primary infection sites. This tissue-specific distribution may be attributed to the differential expression of four CAR splice variants (CAR1–CAR4), which exhibit distinct expression patterns across tissues and developmental stages.

To better understand the CAR’s role in CV-B3 myocarditis and encephalitis pathogenesis, we evaluated CV-B3 infection progression in Balb/c mice across age groups ranging from 7 to 30 days post-birth, with a particular focus on the cardiac system and the central nervous system (CNS). We found that CAR splice variant expression patterns exhibited pronounced tissue tropism with significant age-related variations. During infection, CV-B3 proliferation in young mice (with high CAR1 and CAR2 expression) was accelerated, inducing tissue destruction and inflammation, while it was markedly reduced in 30-day-old mice (with high CAR3 and CAR4 expression). These data demonstrate the underlying susceptibility and severity of CV-B3 infections in the early life of Balb/c mice.

## 2. Results

### 2.1. Seven-Day-Old Balb/c Mice Are Highly Susceptible to CV-B3, Resulting in a Significant Incidence of Severe Illness

**Figure 1:**
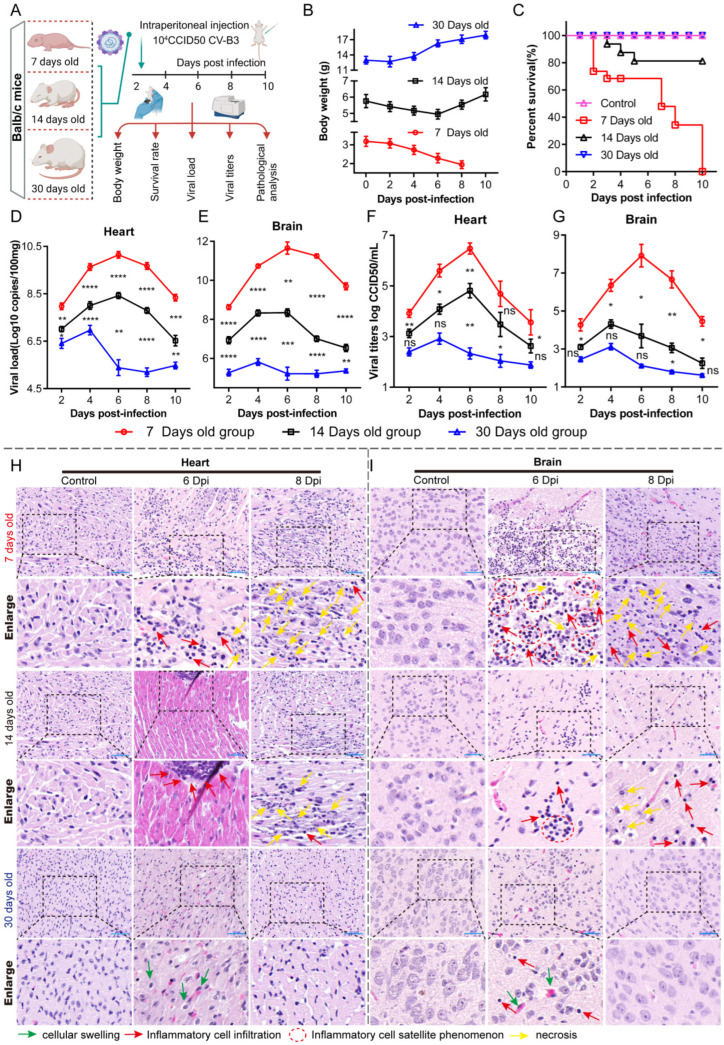
Comparison of the infection characteristics of CV-B3 in 7-, 14-, and 30-day-old Balb/c mice. (**A**) Schematic presentation of comparison and analysis of the characteristics of CV-B3 infection in Balb/c mice across various age groups. (**B**) Weight changes post-CV-B3 infection in Balb/c mice across various age groups (*n* = 15 per group). (**C**) Survival rates of CV-B3-infected Balb/c mice of various ages (*n* = 15 per group). (**D**,**E**) Changes in viral loads in the heart and brain tissues of Balb/c mice across various age groups after CV-B3 infection (*n* = 3 per group). (**F**,**G**) Viral titers in the heart and brain tissues of Balb/c mice infected with CV-B3 across various age groups (*n* = 3 per group). (**H**,**I**) HE staining was used to detect the pathological alterations in the heart and brain tissues of Balb/c mice across various age groups after CV-B3 infection (scale bar: 50 μm). Green arrows indicate cellular swelling, red arrows denote inflammatory cell infiltration, red dashed circles highlight the inflammatory cell satellitosis phenomenon, and yellow arrows mark areas of necrosis) (* *p* < 0.0332, ** *p* < 0.0021, *** *p* < 0.0002, **** *p* < 0.0001; ns, no significant difference).

We established a CV-B3 infection model in mice aged 7, 14, and 30 days by administering 10^4^ CCID50 of CV-B3 via intraperitoneal injection. We monitored body weights and survival rates on days 2, 4, 6, 8, and 10; measured viral titers and viral loads; and performed hematoxylin and eosin (HE) analyses on collected organs (Figure 1A). The 30-day-old mice exhibited continuous weight gain post-infection, while the weight of the 14-day-old mice initially decreased until day 6 and then increased gradually. In contrast, the weight of the 7-day-old mice declined continuously (Figure 1B). There were no fatalities in the 30-day-old mice group, whereas the 14-day-old mice group had an 81% survival rate, and the 7-day-old mice group showed 100% mortality (Figure 1C). In the liver, spleen, lungs, kidneys, and pancreas, the viral load slightly increased within the first 2 days and then declined rapidly in all groups. However, in the heart and brain, the viral load rapidly increased within the first 6 days in the 7-day-old mice and then maintained elevated levels thereafter (Figure S1). The viral load significantly increased in the heart and brain tissues in the different age groups, with the highest levels observed in the 7-day-old mice, followed by in the 14-day-old mice, and the lowest levels observed in the 30-day-old mice (Figure 1D,E). Likewise, the viral titer results in the different age groups were similar to the viral load results (Figure 1F,G). HE staining was performed to evaluate pathological changes in brain and cardiac tissues. In the cardiac tissues (Figure 1H), the 7-day-old mice showed an increase in immune cell infiltration and initial cardiomyocyte degeneration from 6 to 8 days post-infection (dpi). The 14-day-old mice exhibited milder symptoms, while the 30-day-old mice showed the lowest severity, mainly characterized by cardiomyocyte edema and minimal immune response. In the brain tissues (Figure 1I), the 7-day-old mice showed a significant increase in inflammatory cells around neurons by 6 dpi, leading to histiocytes with indistinct borders and disintegrating nuclei by day 8. The 14-day-old mice showed fewer symptoms, with no significant tissue fragmentation, while the 30-day-old mice exhibited slight immune cell infiltration and histiocyte edema, with no notable pathological changes.

### 2.2. A Rapid Innate Immune Response to CV-B3 Infection Was Observed in 7-Day-Old Mice, as Well as in 14- and 30-Day-Old Mice

**Figure 2:**
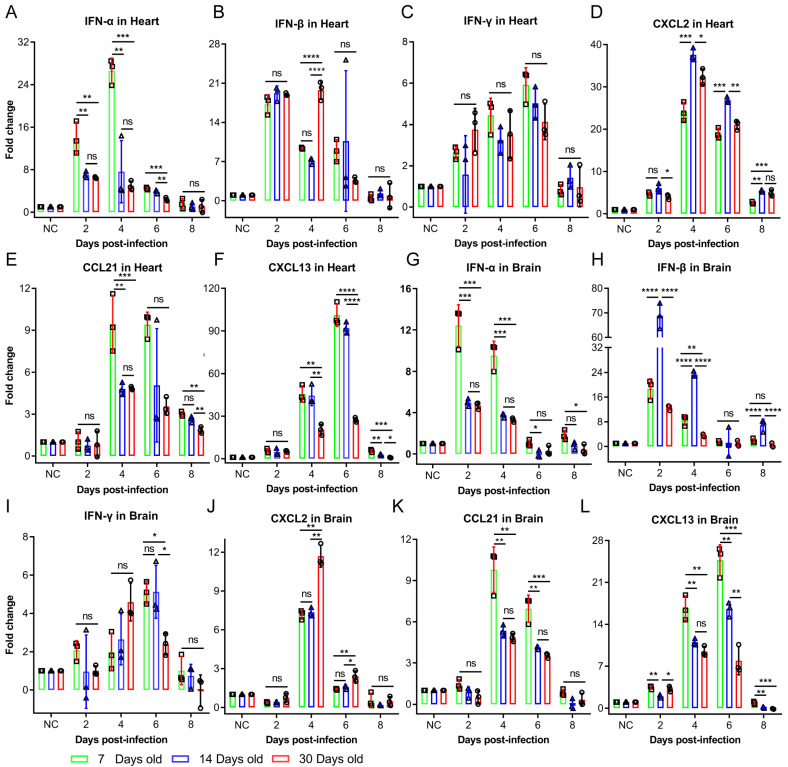
Cytokine levels associated with innate immunity in the heart and brain tissues of 7-, 14-, and 30-day-old mice, related to viral load levels post-CV-B3 infection. (**A**–**F**) IFN-α, IFN-β, IFN-γ, CXCL2, CCL21, and CXCL13 are compared and analyzed in the cardiac tissues of Balb/c mice across different age groups post-CV-B3 infection (*n* = 3 per group). (**G**–**L**) IFN-α, IFN-β, IFN-γ, CXCL2, CCL21, and CXCL13 are compared and analyzed in the brain tissues of Balb/c mice across different age groups post-CV-B3 infection (*n* = 3 per group) (* *p* < 0.0332, ** *p* < 0.0021, *** *p* < 0.0002, **** *p* < 0.0001; ns, no significant difference).

In the early stages of infection (2 to 4 days post-infection), the levels of type I interferons (IFN-α and IFN-β) rapidly increased, with the 7-day-old mice group exhibiting significantly higher IFN-α levels than the 14- and 30-day-old mice groups. Conversely, IFN-β levels were notably elevated in the 14-day-old mice group. After day 6, these cytokine levels returned to normal, with no significant differences among age groups. IFN-γ levels rapidly increased on day 4, peaking on day 6, with no significant differences in cardiac tissues across age groups. However, the brain tissues of the 7- and 14-day-old mice groups had significantly higher IFN-γ levels than those of the 30-day-old mice group (Figure 2A–C,G–I). CXCL2 peaked on day 4 in both brain and heart tissues, with the 14-day-old mice group showing significantly increased levels in cardiac tissue compared to the other age groups (Figure 2D,J). CCL21 also peaked on day 4, with significantly increased levels in the 7-day-old mice group (Figure 2E,K). CXCL13 levels were consistently higher in the 7- and 14-day-old mice groups than in the 30-day-old mice group, peaking on day 6 (Figure 2F,L). Overall, these findings indicate that the increased risk of severe illness and susceptibility to CV-B3 in younger mice may not only be due to differences in immune-related cytokine or chemokine responses.

### 2.3. Splice Variants CAR1 and CAR2 Contributed to Viral Replication Efficiency in CV-B3-Infected Mice

Immunofluorescence data obtained from the heart and brain tissues of the infected mice demonstrated that CV-B3 utilizes CARs to enter these tissues (Figure S2A). An investigation of the correlation between CAR expression levels and viral load across various tissues in the 7-day-old Balb/c mice revealed a positive association between CAR abundance in each organ and early viral load (*p* = 0.028; Figure S2B,C), suggesting that CAR levels may play a significant role in viral proliferation kinetics.

**Figure 3:**
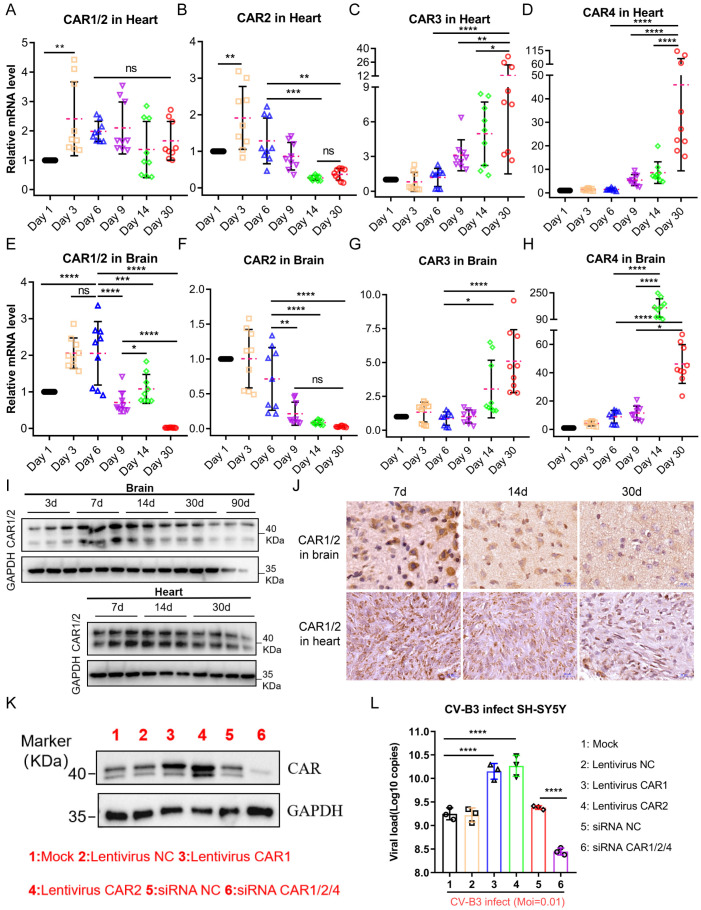
Different expression levels of CAR splice variants in 7-, 14-, and 30-day-old mice, related to viral replication in the heart and brain post-CV-B3 infection. (**A**–**D**) Comparison of the mRNA levels of four distinct CAR gene variants in the heart tissues of Balb/c mice across different age groups (*n* = 9 per group). (**E**–**H**) Comparison of the mRNA levels of four distinct CAR gene variants in the brain tissues of Balb/c mice across various age groups (*n* = 9 per group). (**I**) Western blot assay of CAR1/2 abundance in the brain and heart tissues of Balb/c mice across different age groups. (**J**) Immunohistochemical analysis of the abundance of CAR1/2 in the heart and brain tissues of Balb/c mice across various age groups (scale bar: 20 μm). (**K**) Western blot assay for CAR overexpression and knockdown effects in SH-SY5Y cells. (**L**) Comparison and analysis of viral load 24 h after CAR overexpression and knockdown in SH-SY5Y cells infected with CV-B3 (*n* = 3 per group) (* *p* < 0.0332, ** *p* < 0.0021, *** *p* < 0.0002, **** *p* < 0.0001; ns, no significant difference).

Therefore, we examined CAR expression in mice aged 7 to 30 days and identified four splice variants of the mouse CAR gene (Figure S3). The results indicated that the mRNA levels of the CAR1 and CAR2 splice variants gradually declined with age, whereas those of the CAR3 and CAR4 variants increased (Figure 3A–H). Western blot assays (Figure 3I) and immunohistochemical analyses (Figure 3J) demonstrated that CAR1 and CAR2 protein levels also exhibited a progressive decline over time. To ascertain whether CAR expression levels influence viral proliferation kinetics, we further conducted CAR overexpression and knockdown experiments on SH-SY5Y cells. The findings revealed (Figure 3K,L) that the knockdown group exhibited significantly lower viral loads than the control group, while the CAR overexpression group demonstrated markedly higher viral loads than the control group.

### 2.4. High Virus Loads in the Late Stages of CV-B3 Infection Activate the NLRP3 Inflammasome, Thereby Increasing Proinflammatory Cytokines Such as IL-1β and IL-18

**Figure 4:**
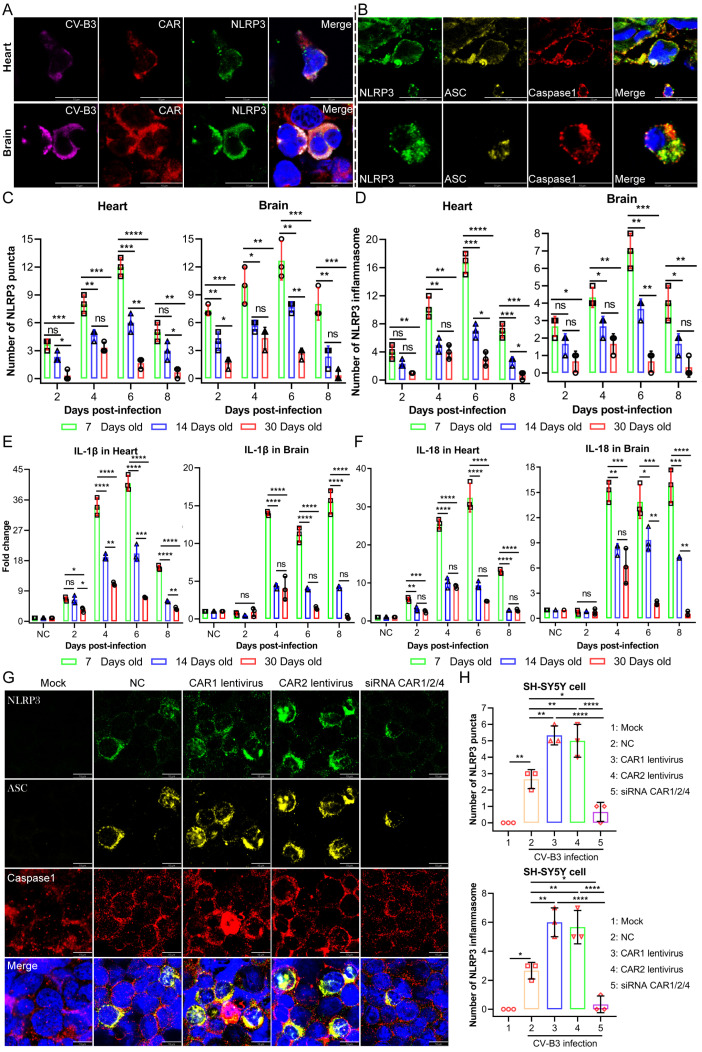
NLRP3 inflammatory vesicle significantly activated in 7-day-old mice post-CV-B3 infection. (**A**) The detection of NLRP3 puncta formation in heart and brain tissues after CV-B3 infection (scale bar: 10 μm). (**B**) The detection of NLRP3 inflammasome assembly in heart and brain tissues post-CV-B3 infection (scale bar: 10 μm). (**C**) Comparison of the quantity of NLRP3 plaques in the brain and heart tissues post-infection with CV-B3 in Balb/c mice across different age groups (*n* = 3 per group). (**D**) A comparative analysis of the levels of NLRP3 inflammatory vesicles in the brain and heart tissues post-infection with CV-B3 in Balb/c mice across various age groups. (**E**) Comparison of IL-1β levels in brain and cardiac tissues post-infection with CV-B3 in Balb/c mice across different age groups (*n* = 3 per group). (**F**) Comparison of IL-18 levels in the brain and heart tissues post-infection with CV-B3 in Balb/c mice across different age groups (*n* = 3 per group). (**G**,**H**) Comparison and analysis of the quantity of NLRP3 plaques and inflammasome in SH-SY5Y cells infected with CV-B3 following CAR knockdown and overexpression (*n* = 3 per group) (scale bar: 10 μm) (* *p* < 0.0332, ** *p* < 0.0021, *** *p* < 0.0002, **** *p* < 0.0001; ns, no significant difference).

In the 7-day-old infected mice, severe pathology was marked by inflammation, with extensive immune cell infiltration, tissue lysis, and fragmentation. This group showed notable NLRP3 inflammasome activation, with increased NLRP3 signaling and inflammasome formation observed in the heart and brain tissues after CV-B3 infection via the CAR (Figure 4A,B). Compared to the 14- and 30-day-old mice, the 7-day-old infected mice group exhibited the highest NLRP3 inflammasome response, and a greater increase was observed in the 14-day-old mice than in the 30-day-old mice (Figure 4C,D). Once the NLRP3 inflammasome assembled, the active Caspase-1 in the inflammasome cleaved Pro-IL-1β and Pro-IL-18, leading to an increase in the levels of the proinflammatory cytokines IL-1β and IL-18, particularly in the 7-day-old mice group, followed by the 14-day-old mice group; however, the increase was not significant in the 30-day-old mice group (Figure 4E,F). In the 7-day-old mice group, increased levels of these cytokines were secreted in the middle and late infection stages; additionally, 60% of fatal cases occurred in this stage, indicating that cytokine overregulation may exacerbate severe inflammation and lead to fatal outcomes.

We previously verified that the abundance of CARs is positively correlated with the regulation of CV-B3 viral proliferation dynamics. Therefore, we further investigated the impact of different viral proliferation dynamics regulated by CAR abundance on the activation level of the NLRP3 inflammasome. We performed CAR overexpression and knockdown experiments on SH-SY5Y cells. After infecting these cells with CV-B3, we observed increased NLRP3 signaling and the formation of the NLRP3 inflammasome. The CAR knockdown group exhibited fewer NLRP3 signals than the control group, while the overexpression group showed a significant increase in NLRP3 signals. Subsequently, the generated NLRP3 signal recruited ASC and Caspase-1 proteins to form the NLPR3 inflammasome. Notably, the CAR overexpression group had a higher number of NLRP3 inflammasomes than the control group, whereas the CAR knockdown group had fewer NLRP3 inflammasomes (Figure 4G,H).

### 2.5. In 7-Day-Old Balb/c Suckling Mice, IL-17-Producing γδT Cells Were Activated Post-CV-B3 Infection

**Figure 5:**
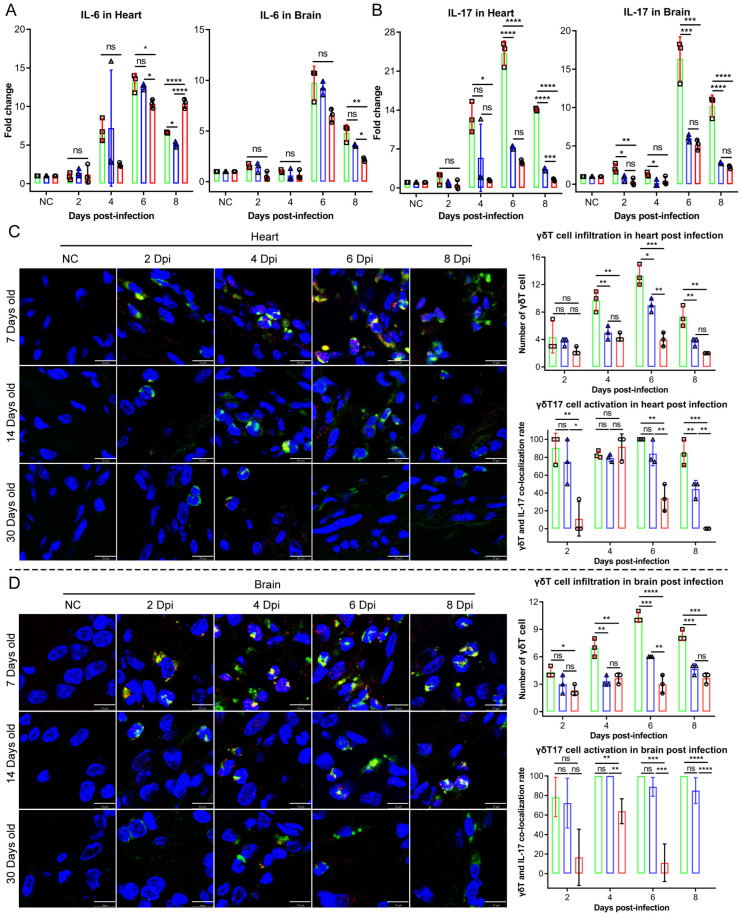
IL-17-producing γδT cells activated in the brain and cardiac tissues post-CV-B3 infection in the 7-day-old mice group. (**A**) Comparison of IL-6 levels in the brain and cardiac tissues post-infection with CV-B3 in Balb/c mice across various age groups. (**B**) Comparison of IL-17 levels in the brain and cardiac tissues post-infection with CV-B3 in Balb/c mice across various age groups (*n* = 3 per group). (**C**) Measurement and comparison of the level of γδT17 cell infiltration and activation in cardiac tissues post-infection with CV-B3 in Balb/c mice across various age groups (*n* = 3 per group) (scale bar: 10 μm). (**D**) Measurement and comparison of the level of γδT17 cell infiltration and activation in brain tissues post-infection with CV-B3 in Balb/c mice across various age groups (*n* = 3 per group) (scale bar: 10 μm) (* *p* < 0.0332, ** *p* < 0.0021, *** *p* < 0.0002, **** *p* < 0.0001; ns, no significant difference).

IL-17 was also found to be one of the most significantly elevated cytokines in the 7-day-old infected mice (Figure 5B). Several studies about the cardiac pathogenesis associated with CV-B3 have revealed that the infiltration of γδT17 cells and their activated production of IL-17 play a pivotal role in driving inflammatory pathology [15]. Furthermore, we observed that variations in CAR expression levels across different age groups significantly impacted the infiltration and activation of γδT17 cells. This is particularly relevant given that the CAR functions as a cell adhesion molecule, which is crucial for facilitating the migratory activation of immune cells during the immune response.

Our immunofluorescence analyses provide compelling evidence that the infiltration and activation of γδT17 cells were most pronounced in the brain and heart tissues of the infected 7-day-old mice (Figure 5C,D). This suggests a potential age-related susceptibility to CV-B3 infection, where the CAR’s role in immune cell migration may influence the severity of the inflammatory responses in these tissues. Overall, these findings indicate the importance of CAR expression in modulating immune cell dynamics and the inflammatory response during viral infections.

## 3. Discussion

The dynamic interplay between viral infection and host immunity critically determines infection outcomes [16]. In this study, the intraperitoneal challenge of Balb/c mice with identical CV-B3 doses revealed significantly enhanced susceptibility and pathological severity in 7-day-old mice compared to in 14- and 30-day-old mice. Cytokine profiling demonstrated the robust activation of both innate and adaptive immune responses in younger infected groups—occasionally exceeding the responses in older groups. However, a cytokine analysis alone does not provide sufficient mechanistic insight into the heightened CV-B3 vulnerability and accelerated disease progression observed in neonates. Viral tropism and replication kinetics are governed by multifactorial determinants [17]. For coxsackieviruses, receptor-mediated entry represents a critical initial step for cellular infection. We therefore quantified CAR expression in the tissues of 7-day-old infected mice, identifying a positive correlation between CAR abundance and early-stage viral loads. Notably, the murine CAR exhibited developmentally regulated isoform expression with tissue-specific distribution patterns. This suggests that age-dependent disparities in CAR expression profiles within target organs (particularly cardiac and CNS tissues) may fundamentally modulate CV-B3 infection efficiency and replication dynamics across developmental stages.

Our analysis revealed an age-dependent decline in the cardiac and cerebral expression of CAR1 and CAR2 splice variants—functional receptors for CV-B3—concomitant with an increased expression of the non-receptor isoforms CAR3 and CAR4 in Balb/c mice. Due to the lack of human samples to verify this phenomenon, there are no data showing whether similar expression patterns exist in human CARs; however, clinical studies using CV-B3 as an oncolytic agent have reported marked CAR upregulation in tumor tissues versus low expression in normal adult tissues [18,19], suggesting the potential conservation of this developmental regulation. This phenomenon is consistent with the role of CAR as an adhesion molecule, essential for intercellular communication during development. The upregulation of CAR4, characterized by an incomplete extracellular domain but a complete intracellular domain, may serve to maintain intercellular signaling functions as the organism matures. Consequently, this phenomenon could be explained by the CAR gene’s involvement in the organism’s developmental biology through variable splicing. However, this regulatory phenomenon impacts CV-B3 infection differently in Balb/c mice of various ages. In younger mice, the high CAR abundance facilitates rapid viral proliferation, allowing for significant “primitive accumulation” during the early “window period” of infection when the immune system is not yet fully activated. In contrast, older Balb/c mice exhibit slower viral proliferation due to their lower CAR abundance.

Upon the detection of endogenous danger signals and exogenous pathogens, the NLRP3 inflammasome—a critical component of innate immunity—rapidly activates to mediate the maturation of the proinflammatory cytokines IL-1β and IL-18. This process induces pyroptotic cell death, releasing inflammatory mediators that recruit additional innate immune cells and establish localized inflammatory responses [20]. While moderate NLRP3 activation supports host defense against microbial threats, excessive activation drives dysregulated inflammation and cytokine storms, which potentiate inflammatory pathologies [21]. In our study, moderate NLRP3 inflammasome activation exerted protective antiviral effects without causing significant tissue damage in the 14- and 30-day-old infected mice groups. Conversely, the 7-day-old infected mice group exhibited NLRP3 hyperactivation, which provided antiviral benefits while simultaneously provoking excessive inflammatory pathology. These findings establish NLRP3 inflammasome dysregulation as a critical determinant of CV-B3-induced tissue damage. In addition to IL-1β and IL-18, serum levels of the proinflammatory cytokine IL-17 were significantly elevated in the 7-day-old infected group. IL-17 is primarily produced by Th17, NK, and γδT17 cells. γδT17 cells have been implicated in the pathogenesis of multiple viral infections. In a hepatitis B virus infection model, these cells exacerbated disease progression by inducing the recruitment of immunosuppressive polymorphonuclear neutrophils (PMNs), thereby promoting CD8^+^ T cell exhaustion [22]. Similarly, skin-resident γδT17 cells were associated with inflammatory tissue pathology in an epicutaneous vaccinia virus infection model [23]. With γδT17 cells being the predominant source following CV-B3 infection, Vγ4^+^ γδT17 cells have been reported as pathogenic mediators in coxsackievirus B3 infection, triggering acute pancreatitis through IL-17 production [15,24]. In healthy organisms, γδT17 cells predominantly reside in peripheral blood, skin, and mucosal-associated lymphoid tissues. Following infection or tissue injury, these cells rapidly infiltrate affected sites. The cell adhesion molecule coxsackievirus and adenovirus receptor (CAR) facilitates immune cell migration to infection/injury loci, and the CAR directly engages junctional adhesion molecule-like protein (JAML) on γδT17 cells, thereby inducing IL-17 release [25,26,27,28]. Based on prior studies, we aimed to assess whether CAR abundance influences the recruitment and activation of γδT17 cells in Balb/c mice of varying ages. Our findings showed that the 7-day-old infected group exhibited the highest CAR abundance, resulting in a significantly increased infiltration and activation of γδT17 cells in cardiac and brain tissues compared to in those of the 14- and 30-day-old mice groups. Overall, CAR abundance critically influences susceptibility to coxsackievirus B3 (CV-B3) infection and the heightened incidence of severe illness in younger individuals. During the early life stages, elevated CAR levels facilitate rapid viral replication during the initial, rate-limiting step of infection. This triggers an immune response characterized by NLRP3 inflammasome overactivation and a substantial production of proinflammatory cytokines, including IL-1β and IL-18. Furthermore, the CAR promotes the migration and activation of γδT17 cells, resulting in increased IL-17 production within cardiac and brain tissues. While the enhanced expression and secretion of these proinflammatory cytokines mediate partial antiviral effects, leading to a decline in viral load and titers, they simultaneously contribute to significant inflammatory pathology. Given that the CAR functions as a universal viral receptor for CV-B and adenovirus—pathogens that are prevalent in younger populations—this study provides novel insights into the heightened susceptibility to CV-B infection during early life and its associated severe disease rates.

This study has several limitations. First, rapid age-dependent changes in CAR alternative splicing occur postnatally, whereas viral-mediated knockdown or overexpression requires extended timeframes for implementation. Second, current viral delivery tools lack tissue specificity for cardiac and brain compartments in neonatal Balb/c mice, impeding targeted CAR manipulation in these critical organs. Finally, as the CAR plays essential physiological roles in cell adhesion and intercellular signaling, constitutive global knockout via gene editing may induce confounding developmental effects. Consequently, we were unable to definitively establish this mechanism in vivo using conventional approaches. Further mechanistic validation could be achieved using cardiac and brain organoids engineered for CAR overexpression or knockdown, coupled with an analysis of CVB3 viral kinetics post-infection.

## 4. Materials and Methods

### 4.1. Animal Models

Balb/c mice, a well-established model for CV-B3 infection studies, were utilized in this study. To analyze age-dependent infection characteristics, 7-, 14-, and 30-day-old mice were selected. The animals were procured from the Laboratory Animal Center, Institute of Medical Biology, Chinese Academy of Medical Sciences. All experimental procedures received prior approval from the Institutional Animal Care and Use Committee of IMBCAMS (Ethical Approval Code: DWSP202308001; date: 8 August 2023) and strictly adhered to the National Institutes of Health Guide for the Care and Use of Laboratory Animals.

### 4.2. Procedure for Animal Experiments

Mice from each age cohort were randomly allocated to two experimental groups: (1) a PBS control group (50 μL, intraperitoneal injection) and (2) a CV-B3-infected group (10^4^ CCID50 of CV-B3 in 50 μL PBS, administered via an intraperitoneal injection). Body weights were recorded using an electronic analytical balance at 2, 4, 6, 8, and 10 days post-infection. Survival rates were analyzed using a GraphPad Prism 8.0.1 survival curve analysis. At predetermined time points (2, 4, 6, 8, and 10 dpi), randomly selected mice were euthanized for organ collection. The harvested tissues were subjected to further analyses, including viral titer determination, viral load quantification, and HE staining.

### 4.3. Virus Strains

The CV-B3 strain HX03/YN/2021 (GenBank accession: PQ001506.1), originally isolated from a clinical rectal swab specimen obtained in Yunnan Province, China, was serially passaged three times in Vero cells to generate the P3 working stock. Virus-containing supernatants were harvested at peak cytopathic effect (>90% CPE). Cellular debris was removed via centrifugation at 2000× *g* for 30 min at 4 °C. Clarified supernatants were concentrated 5-fold using centrifugal filters with a 100 kDa molecular weight cut-(Merck Millipore, Darmstadt, Germany). Viral stocks were titrated by performing a 50% tissue culture infectious dose (TCID_50_) assay on Vero cells, aliquoted, and cryopreserved at −80 °C in Dulbecco’s modified Eagle medium (DMEM) supplemented with 10% fetal bovine serum (FBS).

### 4.4. HE Staining

At 2, 4, 6, 8, and 10 days post-infection, hearts and brains were harvested from the infected Balb/c mice. The tissues were immediately fixed in 10 volumes of 10% neutral buffered formalin at 4 °C for 24 h to ensure complete penetration. After fixation, the specimens were dehydrated through a graded ethanol series (70% → 95% → 100%), cleared in xylene, and infiltrated with paraffin using an automated tissue processor (Leica, Wetzlar, Germany, ASP300). The paraffin-embedded tissues were sectioned at 4 μm thickness with a rotary microtome (Leica RM2245). The tissue sections were deparaffinized in xylene (5 min, 2 times), rehydrated through a graded ethanol series (100% → 95% → 70%; 2 min per concentration), and rinsed in distilled water. Nuclear staining was performed with hematoxylin (Solarbio, Beijing, China, G1120; 8 min), followed by differentiation in 1% acid ethanol (30 s) and bluing in 0.2% ammonia water (1 min), and the sections were then rinsed under running tap water for 30 min. Cytoplasmic counterstaining was conducted with eosin (Solarbio, G1100; 1 min), followed by dehydration through graded ethanol (95% → 100%; 1 min per concentration) and clearing in xylene (5 min, 2 times). Finally, the sections were mounted with neutral balsam. Whole-slide imaging was performed using a Pannoramic MIDI digital slide scanner (3D HISTECH, Thermo Fisher Scientific, Waltham, MA, USA). A histopathological analysis was conducted using CaseViewer 2.4.0.

### 4.5. Western Blotting Assay

Immediately after euthanasia, the heart and brain tissues (20 mg) obtained from the Balb/c mice were flash-frozen in liquid nitrogen and stored at −80 °C. For protein extraction, the tissues were minced on ice and homogenized in 300 μL ice-cold RIPA lysis buffer, which was supplemented with 1× protease inhibitor cocktail and 1× phosphatase inhibitor cocktail. Following complete lysis in RIPA buffer, tissue/cell homogenates were centrifuged at 12,000× *g* for 30 min at 4 °C, and supernatants were quantified using a Pierce™ BCA Protein Assay Kit (Thermo Fisher Scientific, 23225). Equal amounts of total protein were denatured in 5× SDS loading buffer at 95 °C for 10 min, then the denatured samples were resolved via SDS-PAGE on 12% gels and transferred to 0.22 μm PVDF membranes (Merck Millipore, IPFL00010) using a Trans-Blot^®^ SD Semi-Dry Transfer System (Bio-Rad, Hercules, CA, USA), and the membranes were blocked with 2% BSA in PBST (0.1% Tween-20) for 1 h at 25 °C. Primary antibodies were diluted in blocking buffer and incubated overnight at 4 °C (Rb mAb to CAR/CXADR (ABclonal, Wuhan, China, A22607), Mouse mAb to CV-B3 Merck MAB948, Rb mAb to NLRP3 (Abcam, Cambridge, UK, Ab210491), Rb mAb to Caspase-1(Proteintech, Chicago, IL, USA, 22915-1-AP), Mouse mAb to ASC (SANTA Cruz, CA, USA, sc-514414), Mouse mAb to HRP&GAPDH (Proteintech, HRP-60004). After three washes with PBST (5 min each time), the membranes were incubated with HRP-conjugated secondary antibodies (Goat Anti-Rabbit IgG H&L (HRP) Abcam ab6721, Goat Anti-Mouse IgG H&L (HRP) Abcam ab6789) for 1 h at 25 °C. Protein bands were visualized using BIO-RAD ChemiDoc.

### 4.6. Immunohistochemistry (IHC)

After deparaffinization in xylene and rehydration through graded ethanol, antigen retrieval was performed in pH 6.0 citrate buffer (95 °C, 20 min). Endogenous peroxidase activity was quenched with 3% H_2_O_2_ (25 °C, 10 min). Sections were blocked with 5% normal goat serum (25 °C, 30 min) and incubated with rabbit anti-CAR/CXADR monoclonal antibody (GeneTex, Irvine, CA, USA, GTX118382; 1:300) at 4 °C overnight, followed by response enhancer treatment (Solarbio, SP0021) and secondary antibody incubation (Solarbio, SP0021; 25 °C, 30 min). Immunoreactivity was visualized using DAB substrate (Solarbio, DA1010; 5 min at 25 °C). Nuclei were counterstained with hematoxylin (Solarbio, G1120; 8 min), followed by differentiation in 0.5% acid ethanol (30 s) and bluing in tap water substitute (1 min). Following dehydration through graded ethanol and xylene clearance, the sections were mounted with neutral balsam. Whole-slide images were acquired at 20× magnification using a Pannoramic MIDI slide scanner (3D HISTECH) and analyzed using CaseViewer 2.4.0.

### 4.7. Immunofluorescence (IF)

The NLRP3 inflammasome is a multiprotein complex that serves as a critical platform for innate immune activation. In quiescent cells, NLRP3 exhibits low expression levels and diffuse cytoplasmic localization. Upon stimulation, NLRP3 undergoes transcriptional upregulation and oligomerizes into discrete punctate foci. These structures subsequently recruit the adaptor protein ASC and pro-Caspase-1, culminating in the assembly of the functional NLRP3 inflammasome complex. The formation of NLRP3 puncta represents a morphological hallmark of inflammasome activation, providing the structural basis for Caspase-1 autocatalysis. Activated pro-Caspase-1 then proteolytically processes the proinflammatory cytokines pro-IL-1β and pro-IL-18 into their bioactive forms (IL-1β and IL-18), which are secreted extracellularly. Concurrently, Caspase-1 triggers pyroptosis—a lytic, proinflammatory mode of programmed cell death. Crucially, the co-localization of NLRP3 puncta with ASC and Caspase-1 constitutes definitive evidence of functional inflammasome assembly and signifies the initiation of a potent inflammatory cascade within the affected cell.

Following dewaxing in xylene (2 times, each time 5 min) and rehydration through graded ethanol (100% → 95% → 70%; 2 min at each concentration), tissue sections were subjected to antigen retrieval in citrate buffer (10 mM, pH 6.0; 95 °C, 20 min), endogenous peroxidase blockade with 3% H_2_O_2_ (10 min, 25 °C), and permeabilization with 0.3% Triton X-100 (15 min, 25 °C) prior to blocking in 5% BSA (1 h, 25 °C). The primary antibodies were incubated overnight at 4 °C: Mouse anti-CV-B3 VP1 (Merck MAB948; 1:300), Rabbit anti-CAR/CXADR (ABclonal A22607; 1:500), Rat anti-NLRP3 (Thermo Fisher Scientific, MA5-23919; 1:500), Rabbit anti-Caspase-1 (Proteintech 22915-1-AP; 1:500), Mouse anti-ASC (Santa Cruz, sc-514414; 1:500), Armenian hamster anti-γδTCR (BioLegend, San Diego, CA, USA, 118108; 1:500), and Rat anti-IL-17A (BioLegend 506916; 1:500). Following PBST washes, species-matched secondary antibodies were applied for 1 h at 25 °C in darkness: Goat anti-rat IgG-Alexa Fluor 488 (Abcam ab150157; 1:500), Goat anti-mouse IgG-Alexa Fluor 555 (Abcam ab150118; 1:1000), Donkey anti-rabbit IgG-Alexa Fluor 647 (Abcam ab150063; 1:1000), and Goat anti-Armenian hamster IgG-Alexa Fluor 647 (Abcam ab173004; 1:500). Nuclei were counterstained with DAPI, slices were mounted, and confocal images were acquired using a Leica TCS SP8 laser confocal microscope.

### 4.8. ELISA Analysis

Cardiac and cerebral tissues were minced in 2 mL microtubes using sterile ophthalmic scissors, enzyme-free stainless-steel beads were added, and homogenization was performed with 100 μL ice-cold PBS in a homogenizer (60 Hz, 10 × 30 s cycles with 30 s ice-cooling intervals between cycles). Homogenates were centrifuged at 10,000× *g* for 10 min at 4 °C, and supernatants were collected. IFN-α, IFN-β, IFN-γ, CXCL2, CCL21, and CXCL13 concentrations were quantified using commercial ELISA kits, according to the manufacturers’ protocols.

### 4.9. Quantitative Real-Time PCR

Viral RNA was extracted using TRIzol (absin, Shanghai, China, abs9331) following the manufacturer’s protocol, including chloroform phase separation and isopropanol precipitation. The RNA purity and concentration were verified using Thermo Fisher Scientific NanoDrop™ spectrophotometry (A260/A280 > 1.8). TaqMan probe-based qPCR for CV-B3 viral load was performed with a One Step PrimeScript™ RT-PCR Kit (Takara, Ōtsu, Shiga, Japan, RR064A) on an ABI 7500 Real-Time PCR System (Applied Biosystems, Foster City, CA, USA), according to the manufacturer’s procedure. The primer set is shown in Table 1. Viral copy numbers were calculated as a ratio with respect to the standard control (the formula used to calculate the standard copy number was as follows: copies/mL = 6.02 × 10^23^ (copies/mol) × concentration of standard (g/mL)/RNA base number × 340 g/mol).

Viral load standards were prepared as described below: a 852 bp fragment (Supplementary Table S1) encompassing the CV-B3 (GenBank accession: PQ001506.1) VP1 gene was cloned into the pUC57 vector. The recombinant plasmid was linearized with ECORI and purified using 1.2% agarose gel and a PCR Clean-up Kit (Macherey-Nagel, Düren, Germany, 740609). In vitro transcription was performed with a T7 kit (TaKaRa, Code No. 6140), followed by DNase I treatment to purify the RNA. Ten-fold serial dilutions (10^7^ to 10^0^ copies/μL) of the purified RNA standard were prepared, and each dilution was analyzed in quadruplicate using a One Step PrimeScript™ RT-qPCR Kit (Takara, RR064A) on an ABI 7500 Real-Time PCR System (Applied Biosystems). The standard curve was generated by plotting the quantification cycle (Cq) values against the log_10_ input copy numbers. A linear regression analysis yielded the following equation (Supplementary Figure S1A): y = −3.3609x + 43.855 (where y = Cq and x = log_10_ (copies/μL), with slope = −3.3609 and intercept = 43.855).

The total RNA was extracted from the Balb/c mice heart and brain tissues using TRIzol reagent. Quantification of the relevant expression levels of the four splice variants of the Balb/c mouse CAR gene was performed using the comparative ΔΔCq method with SYBR Green-based real-time PCR (Takara, RR066A). Gene expression levels were normalized to the endogenous reference gene GAPDH, and fold changes were calculated relative to the control group. The primer sequences are provided in Table 1.

### 4.10. Virus Titer Assay

The heart and brain tissues were placed in 1.5 mL tubes, minced with fine surgical scissors, and suspended in 100 μL of 1× PBS. After tight sealing, the samples were stored at −80 °C overnight. The frozen tissues were thawed at 25 °C and subjected to three freeze–thaw cycles with thorough grinding. Lysates were centrifuged at 10,000× *g* for 10 min at 4 °C. Supernatants containing virus particles released during the freeze–thaw cycles were serially diluted (10^−1^ to 10^−7^) in MEM maintenance medium supplemented with 2% FBS. Aliquots (100 μL) of each dilution were incubated with 10^4^ Vero cells/well in 96-well plates at 37 °C under 5% CO_2_. CPEs were monitored daily for 7 days, with the experimental endpoint defined as the timepoint showing no further CPE progression. Viral titers were calculated as TCID_50_/mL using the Reed–Muench method.

### 4.11. Statistical Analysis

All statistical analyses were conducted and all plots were constructed using GraphPad Prism software. All experimental data are presented as the mean ± standard deviation (SD). A minimum of three independent biological replicates were analyzed per experimental group (*n* ≥ 3). A one-way ANOVA with Tukey’s multiple comparisons test was performed, with statistical significance defined as *p* < 0.05. *p*-values are reported as follows: GP: 0.1234 (ns), 0.0332 (*), 0.0021 (**), 0.0002 (***), 0.0001 (****).

## Figures and Tables

**Table 1:** Primers used in this study.

Primer	Sequence
CV-B3-F	GGCAGACATCCACTAACCCT
CV-B3-R	TTTCTGGCAAATTCTGACCAACC
CV-B3-probe	ATGCACCGCCACGTATGTCC
M-CAR1-F	AAGTCTGGCGACGCATCTAT
M-CAR1-R	GGTTCACATTTTAGCTTGAAGTC
M-CAR2-F	CCAGGAGCTATATTGGCAGCA
M-CAR2-R	TAATGCCATCGGTCTTGTA
M-CAR3-F	ACGATGTCAAGTCTGGCGAC
M-CAR3-R	CCTGCCACCTTGTAACTCCG
M-CAR4-F	TGGCGGCCTCTCAAGGACAGA
M-CAR4-R	ATGGCGTAGGCATTGTCTGG
M-GAPDH-F	ACTCTTCCACCTTCGATGCC
M-GAPDH-R	TGGGATAGGGCCTCTCTTGC

## Data Availability

All datasets used and/or analyzed during the current study are available from the corresponding author on reasonable request.

## Supplementary Materials

The following supporting information can be downloaded at: https://www.mdpi.com/article/10.3390/ijms26157163/s1.

## Abbreviations

The following abbreviations are used in this manuscript:

CV-B3	coxsackievirus B3
HFMD	hand, foot, and mouth disease
CAR	coxsackievirus adenovirus receptor
dpi	days post-infection
CPE	cytopathic effect
ASC	apoptosis-associated speck-like protein containing CARD
NLRP3	NOD-like receptor family pyrin domain containing 3
Caspase1	cysteinyl aspartate specific proteinase 1
TCID_50_	50% tissue culture infectious dose
HE	hematoxylin and eosin
DMEM	Dulbecco’s modified Eagle medium
FBS	fetal bovine serum
