# Supplementary material for: The Expression Pattern of the Splice Variants of Coxsackievirus and Adenovirus Receptor Impacts CV-B3-Induced Encephalitis and Myocarditis in Neonatal Mice
Source: Int J Mol Sci. 2025 Jul 24;26(15):7163. doi: 10.3390/ijms26157163 (PMC12346337; doi:10.3390/ijms26157163)
Supplement: Supplementary file 1 [file ijms-26-07163-s001.zip › ijms-3738795-supplementary.pdf]

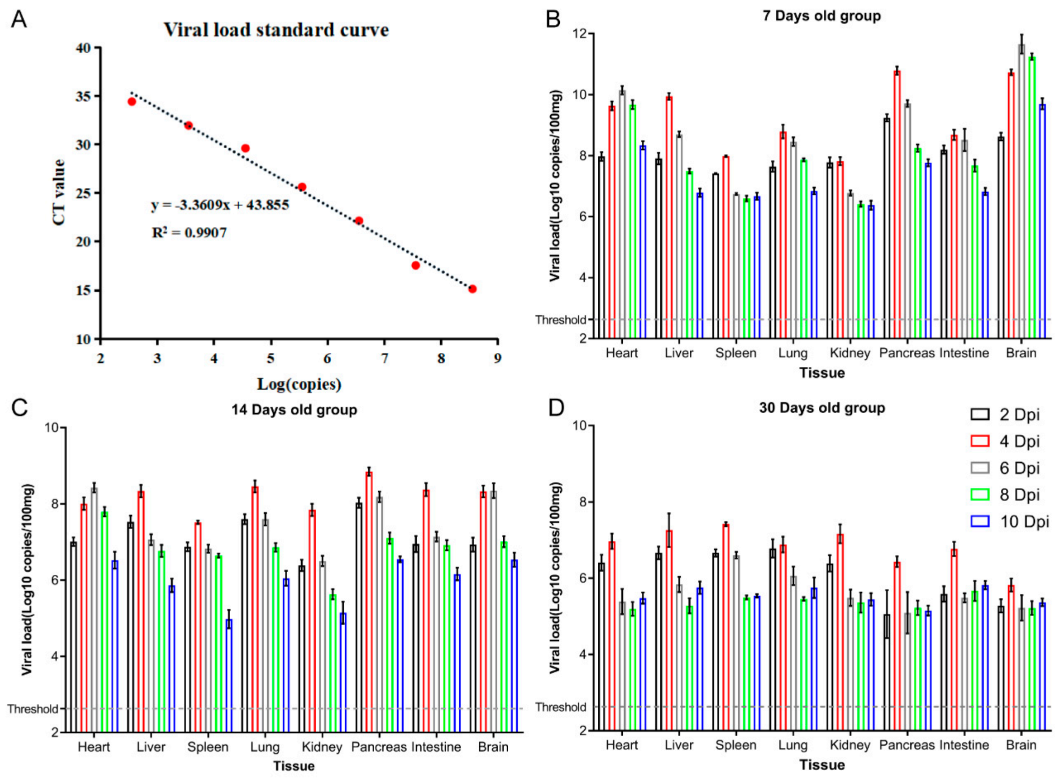

Figure S1. Viral load in various tissues and organs of Balb/c mice of various ages following CV-B3 infection. (A) A standard curve for CV-B3 viral load. (B-D) Viral load in various tissues and organs of Balb/c mice infected with CV-B3 at 7 days (B), 14 days (C), and 30 days aged mice (D).

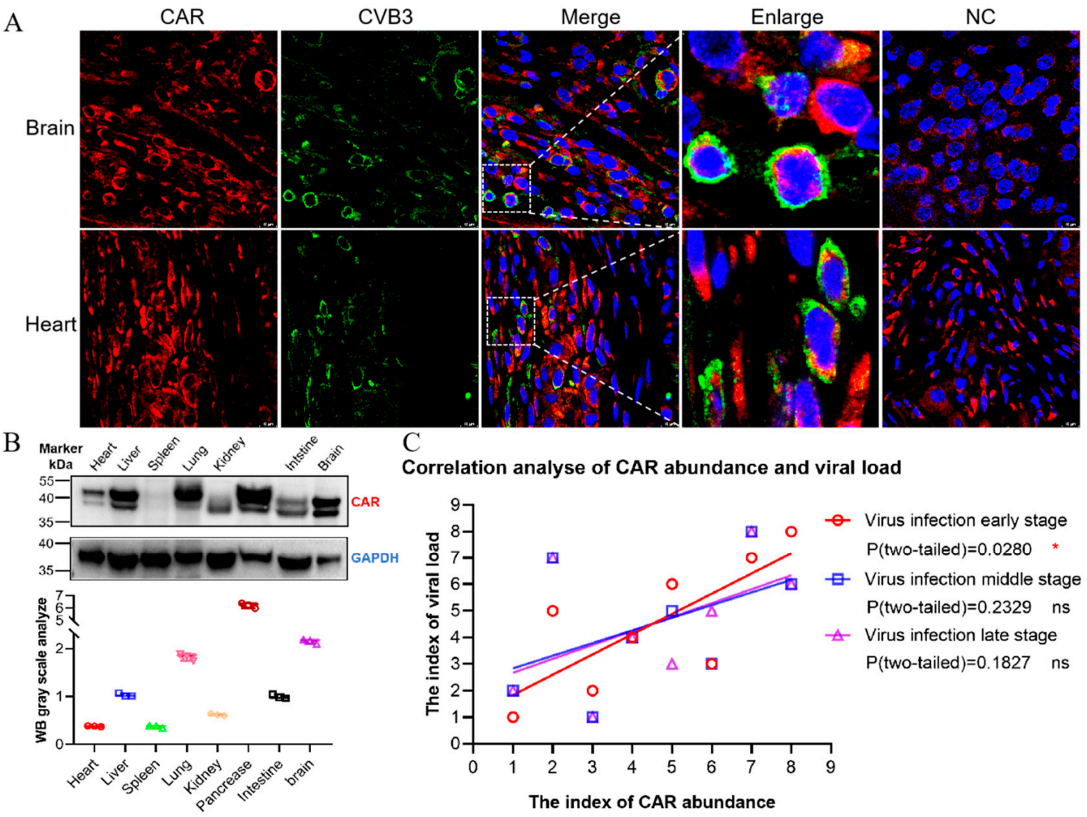

Figure S2. Analysis of the correlation between the abundance of CAR receptor expression in tissues and organs and the viral load at different stages of infection. (A) Immunofluorescence colocalization of CV-B3 and CAR receptors in cardiac and brain tissues. (B) Western Bolt detects the abundance of

CAR receptors in various tissues and organs of Balb/c mice. (C) Analysis of the correlation between the abundance of CAR receptor expression in tissues and organs of 7-day-old Balb/c mice and viral load at different stages of infection.

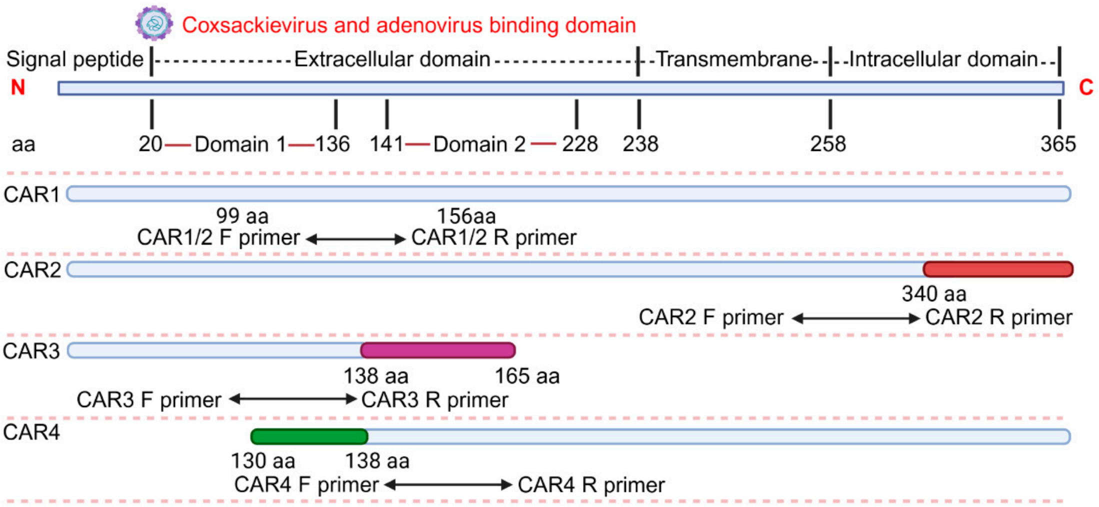

Figure S3. The diagram shows the amino acid sequence variance of the four splice variants of the Balb/c mouse CAR gene.

Table S1. CV-B3 viral load RNA standard sequence.

**CV-B3 Viral Load RNA Standard Sequence**
GGGCCGGTTGAGGATGCAGTGACGGCCGCGATTGGCAGGGTCGCCGACACCATAGGGACTG-GACCAACAAATTCTGAGGCCATACCGGCTCTTACAGCAGCTGAGACTGGGCACACATCCCAAGTTGTCCCAGGTGACACCATGCAAACGC GTCACGTGAAGAACTATCACTCGAGATCTGAGTCGACAATTGAGAACTTCGTGTG-CAGGTCTGCGTGTGTTTATTTTACAGAGTACGAGAACTCAGGATCAAATCGGTATGCTGAATGGGTGATAACAACCCGCCAAGCAGTACA GTTGAGAAGAAAGTTGGAGTTCTTCACATACATGAGGTTTGATTTAGAGCTCAC-CTTCGTTATTACTAGTACTCAACAACCCTCTACAACCCAGAACCAAGACGCCCAGATTCTCACACATCAGATAATGTATGTGCCACCAGGT GGTCCGGTACCAGATAAGGTTGACTCATACGTGTGGCAGACATCCACTAACCCTAG-TGTCTTCTGGACCGAAGGCAATGCACCGCCACGTATGTCCATTCCATTCTTGAGCATTGGCAATGCATATTCCAACTTTTATGACGGTTGGT CAGAATTTGCCAGAAACGGGGTATATGGCATAAATACCCTGAACAATATGG-GAACCCTTTATGCAAGACACGTGAATGCTGGGAGCACAGGACCAATAAAAAGCACCATCAGAATCTATTTTAAACCAAAGCATGTCAAG GCTTGGATACCTAGGCCTCCAAGGTTGTGTCAGTATGAGAAA-GCAAAAAATGTTAACTTCCAACCTAGTGGTGTAACGACGACCAGACAGAGCATTACAGCAATGACAAATACTGGTGCATTT
